# Standing at the edge of mortality; Five-year audit of an emergency department of a tertiary care hospital in a low resource setup

**DOI:** 10.12669/pjms.37.3.3680

**Published:** 2021

**Authors:** Sama Mukhtar, Syed Ghazanfar Saleem, Saima Ali, Sarfraz Ahmed Khatri, Anna Q Yaffee

**Affiliations:** 1 Sama Mukhtar, FCPS. Consultant Emergency Department, The Indus Hospital, Karachi, Pakistan; 2 Syed Ghazanfar Saleem, FCPS. Consultant Emergency Department, The Indus Hospital, Karachi, Pakistan; 3 Saima Ali, FCPS. Consultant Emergency Department, The Indus Hospital, Karachi, Pakistan; 4 Sarfraz Ahmed Khatri, FCPS –II Trainee. Resident Emergency Medicine, The Indus Hospital, Karachi, Pakistan; 5 Anna Q Yaffee, MD, MPH. Consultant EM, Grady Memorial Hospital, Emory University, Atlanta, USA

**Keywords:** Emergency department, Pakistan, audit, mortality

## Abstract

**Background & Objective:**

Understanding the demographics of mortality and its burden in the emergency department of a tertiary care setup can lead to better planning and allocation of resources to streamline process flow. This can be achieved systematically through mortality audit that can identify the loopholes and areas of improvement. Our objective was to characterize the epidemiology of ED mortality in a tertiary care hospital of Karachi, Pakistan.

**Methods:**

A five-year retrospective chart review of 322 adult mortalities presenting between January l, 2014 – December 31, 2018 was conducted in the emergency department (ED) of The Indus Hospital (TIH), Karachi. All expiries in ED were included while those brought dead and with do not resuscitate order (DNAR) were excluded.

**Results:**

Mortality incidence of 0.076% (7.6/10,000 ED visits in five years) was reported. Amongst 507,759 adult ED visits, 322 mortalities were documented. Mean time lapse before presentation was 44±147 hours and mean length of stay before death was 3.4±2.8 hours. Acute coronary syndrome (ACS) was the predominant cause of death with 109 (33.8%) expiries. Significant association was reported between no history of prior care and high priority (P1) cases (p=0.013).

**Conclusions:**

This study identified the contributing factors to adverse outcome such as delayed presentation with systemic gaps in management and unknown disposition. The need to improve these factors at local and national level can lead to improvement in Pakistani healthcare sector.

## INTRODUCTION

The role of emergency medicine is to provide care to patients with acute and unexpected fatal illnesses.^1^-^6^ ED mortality is a reflection of healthcare and mortality rates of 0.77/1000 have been reported in USA.^2^ Multiple factors impact clinical outcome, including timely access to emergency care, availability and quality of pre-hospital and hospital management, overcrowding and length of stay in ED and patient factors such as patient age and co-morbidities.^3^ According to World Health Organization (WHO) the top three causes of mortality are stroke, ischemic heart disease (IHD) and trauma.^4^ WHO has highlighted the need to strengthen emergency care capacity of health systems in low and middle income countries (LMIC).^7^ In 2015, 28.3 million deaths globally were attributed to emergency cases. This burden is 4.4 times higher in LMICs but may be reduced by upgrading access to emergency care.^8^,^9^ Emergency medicine has been under-prioritized and is an emerging field in Pakistan with the potential to expend.^5^,^6^

The Indus Hospital (TIH) is a free of cost, private sector hospital; located in Korangi district (population of 2,457,019)^10^ in Karachi; A city with a population of 16,093,786.^11^ TIH ED receives more than 400 patients per day and over 500 deaths are registered annually. With limited literature on the relationship between ED care and subsequent mortality; little is known about trends of ED mortality in Pakistan. Understanding the trend of mortality in EDs will provide information on how measures to intervene upon and prevent these fatalities can be implemented. This study was intended to delineate the epidemiological characteristics of mortalities in our ED with respect to frequency and cause of death. We hope this study will foster future research and lead to development of care pathways, leading to improved patient outcome.

## METHODS

A retrospective chart review of patients, ≥14 years of age, who had expired at TIH ED between 1^st^ January 2014 to 31^st^ December 2018, was performed. Those who were brought dead or with do not resuscitate (DNAR) orders were excluded. Study was exempted from IRB approval under number (IRD_IRB_ 2019_05_001) on May 06, 2019.

Data was extracted via Health Management Information systems (HMIS) and entered on pre-designed questionnaire. Results were de identified and coded into an electronic database by a data analyst. The charts were reviewed by two independent investigators and conflicts were resolved by a third investigator.

Data was reviewed for time to presentation, age, sex, presenting complaints, co-morbidities, prior medical care, medication history and vital signs. Triage acuity, based on the Manchester triage system (P1; immediate, P2; very urgent, P3; urgent) was assessed. Alertness level based on AVPU scale (Alert, response to verbal stimulus, response to painful stimulus, unresponsive) was analysed. The provisional ED diagnosis at death and length of stay in ED was documented.

Descriptive statistics were reported as frequencies and percentages to present categorical data. Continuous data were presented as mean ± standard deviation (SD). Chi-square test was used to test the difference between categorical variables, p <0.05 was considered significant. Data analysis was with SPSS-21. The number of deaths per 10,000 ED visits was calculated over the relevant time period.

## RESULTS

Total ED visits of 707,417 were reviewed. Out these, 199,658 (28.2%) were referred to the TIH Family Medicine clinic directly from triage. The TIH ED treated 507,759 (71.8%) patients, of which 18,953 (3.7%) were admitted in wards. 291,209 patients (57.4%) were successfully discharged from ED and 29,776 (5.9%) patients left against medical advice (LAMA) or were discharged on request (DOR). A total of 56,315 (11%) patients were referred out due to limitations in hospital infrastructure and lack of required specialties. There was undocumented disposition in 109,524 (21.6%) patients.

**Fig.1:**
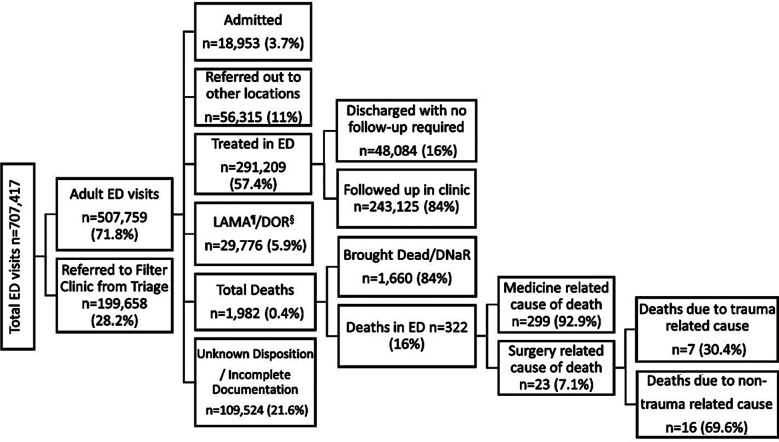
Schematic presentation of Five- Year ED visits (January 01, 2014 – December 31, 2018) Mortality= Deaths in ED/Adult ED visits- (Referred out patients + LAMA).

Out of a total of 1982 (0.4%) deaths in the ED, 1660 (84%) were brought dead or DNAR. Medical records were reviewed for 322 (16%) patients who were resuscitated but subsequently died. The reported mortality incidence was 0.076% (7.6 deaths per 10,000 ED visits in five years. In our study, most mortalities had medical causes 299 (92.9%), while 23 (7.1%) had surgical causes, out of which all trauma mortalities were in male gender 7 (100%). (Fig.1).

**Table-I:** Co-relation of Acuity levels with history of prior care, medication history and AVPU Scale (N = 322).

Trit Triage Acuity level				

	P1 n (%)	P2 n (%)	P3 n (%)	P-value
***History of Prior Care***			
No	117 (64.6%)	55 (53.9%)	12 (38.7%)	0.013
Yes	64 (35.4%)	47 (46.1%)	19 (61.3%)	
***Medication History***			
No	94 (50.5%)	36 (36%)	07 (21.9%)	0.002
Yes	92 (49.5%)	64 (64%)	25 (78.1%)	
***AVPU ^†^ Scale***			
A	60 (32.1%)	89 (87.3%)	32 (97%)	<0.001
V	10 (5.3%)	07 (6.9%)	0 (0.0%)	
P	18 (9.6%)	06 (5.9%)	01 (3.0%)	
U	99 (52.9%)	0 (0.0%)	0 (0.0%)	

AVPU† Scale: A=Alertness, V= response to verbal stimulus, P= response to painful stimulus, U= unconscious.

The mean age at death was 53.9 ± 16.6 years (range 14 - 97 years). Mortality was most frequent 121 (37.6%) in the 55-69-year age group; followed by 84 (26.1%) in 40-54 years, 50 (15.5%) in ≥70 years, 44 (13.7%) in 26-39 years and 23 (7.1%) in 14 -25 years age group. 193 (59.9%) of the deaths were male. The mean time lapse before presentation in ED was 44±147 hours, range 0.25-2160 hours. The mean length of stay in ED before death was 3.4±2.8 hours, range 0.8-23.2 hours.

One hundred eighty seven (58.1%) of patients who died were triaged P1, 102 (31.7%) P2 and 33 (10.2%) were P3 based on Manchester triage system. On the AVPU scale, 99 (52.9%) P1 patients were unconscious on presentation. A significant association was found between patients having history of prior care and triage priority. Those with no history of prior care were high priority (P1) cases (p=0.013). Another significant association was found between the history of medication use and triage priority. Those who had the history of medication use had less severe symptoms, thus on lower priority level (p=0.002).

In terms of provisional causes of deaths, acute coronary syndrome was predominant 109 (33.8%) followed by complications of cardiovascular disease 46 (14.3%) with equal mortality from electrolyte imbalance and sepsis 21 (6.5%). The most common presenting complaint of ED mortality was breathlessness 159 (49.4%), followed by loss of consciousness (LOC) in 64 (19.9%) and chest pain in 49 (15.2%) respectively. The most frequently reported co-morbidity was hypertension 114 (36.4%), diabetes mellitus 85 (27.2%) and Ischemic heart disease (IHD) 67 (21.4%). To enable subgroup analyses, we subdivided our study population according to age. Acute coronary syndrome was the commonest provisional cause of death in patients over 26 years; with highest frequency in the age group 55- 69 years, (n=58, 47.9%). (Table-II).

**Table-II:** Co-morbid condition, presenting complaint and provisional ED diagnosis at the time of death in order of frequency and age groups (N=322).

Age	14-25 years	26-39 years	40-54 years	55-69 years	70 + years
***Co-morbid condition n (%)***				
1.	MAC¶ 4(17.4%)	HTN* 10 (25.6%)	HTN 34 (42%)	HTN 54 (45%)	HTN 15 (30%)
2.	Pulmonary TB 2(8.7%)	IHD‡ 5 (12.8%)	DM† 33 (40.7%)	IHD 36 (30%)	DM 13 (26%)
3.	Thalassemia Major 1(4.3%)	MAC 4 (10.3%)	IHD 15 (18.5%)	DM 35 (29.2%)	IHD 11 (22%)
***Presenting Complaints n (%)***				
1.	Breathlessness 8 (34.8%)	Breathlessness 16 (36.4%)	Breathlessness 45 (53.6%)	Breathlessness 65 (53.7%)	Breathlessness 25 (50%)
2.	Vomiting 4 (17.4%)	LOC§ 8 (18.2%)	LOC 21 (25%)	Chest pain 25 (20.7%)	LOC 12 (24%)
3.	Trauma 3 (13%)	Fever 7 (15.9%)	Chest pain 11 (13.1)	LOC 21 (17.4)	Chest pain 8 (16%)
***Provisional ED diagnosis at the time of death n (%)***				
1.	MAC 5 (21.7%)	ACS** 6 (13.6%)	ACS 24 (28.6%)	ACS 58 (47.9%)	ACS 20 (40%)
2.	Acute abdomen 3 (13%)	Acute abdomen 4 (9.1%)	CVD∞ 14(16.7%)	CVD 20 (16.5%)	CVD 7 (14%)
3.	Trauma 3 (13%)	Sepsis 4 (9.1%)	Sepsis 7 (8.3%)	CVA†† 7 (5.8%)	Metabolic Abnormalities 6 (12%)

* Hypertension.

† Diabetes Mellitus.

‡ Ischemic Heart Disease.

§ Loss of consciousness.

¶ Malignancy associated Complications.

∞ Cardiovascular disease complications.

** Acute coronary syndrome.

†† Cerebro-vascular accident.

Highest mortality was reported between 00:00 to 5:59 hours (n=88, 27.3%). Mortality was consistent throughout 24 hours in P1 and P2 acuity. However, higher mortality was seen in P3 acuity between 18:00 to 23:59 hours (n=10, 13.3%) and 00:00 to 05:59 hours (n=11, 12.5%) as compared to the rest of the day (Table-III).

**Table-III:** Correlation of deaths at different times in a 24-hours shift with triage acuity; in an ED of a tertiary care Hospital, Karachi, Pakistan. (N= 322).

Triage Acuity 00:00 - 05:59 hours 06:00 -11:59 hours		Time of Presentation during 24-hours shift				Total

		12:00-17:59 hours	18:00-23:59 hours	n (%)		
P1	Count	47	45	52	43	187
	% within Time of Presentation	53.40%	60.00%	61.90%	57.30%	58.10%
P2	Count	30	24	26	22	102
	% within Time of Presentation	34.10%	32.00%	31.00%	29.30%	31.70%
P3	Count	11	6	6	10	33
	% within Time of Presentation	12.50%	8.00%	7.10%	13.30%	10.20%
Total % within Time of Presentation	Count	88	75	84	75	322
	100.00%	100.00%	100.00%	100.00%	100.00%	

## DISCUSSION

Pakistan as an LMIC requires effective and structured emergency care. At TIH, Karachi we reported an ED mortality rate of 0.076% (7.6 per 10,000 visits in five years). This figure is lower than anticipated and may reflect a high standard of emergency care, but alternatively could be attributed to critical patients being referred out from the ED after initial stabilization due to unavailability of beds and lack of certain medical and surgical service lines. A large number (n=29776, 5.9%) of patients are LAMA/DOR. The outcome of these patients remains unknown. Local literature reports a mortality rate of 0.7% number,^12^ 10-fold higher than our findings. This finding can pave the way for future planning in expanding infrastructure and specialty care at TIH that currently leads to out of hospital referrals after initial resuscitation.

Our study identified most of the mortalities in 55+ years with a male predominance; consistent with local^13^,^14^ and published literature out of Nepal.^15^ All trauma mortalities were documented in males, in conformity with African literature.^16^,^17^ Majority of death were attributed to medical causes 299(92.9%), in line with UMIC (Upper middle income countries)^18^ and LMIC data.^15^,^16^ A study in Karachi identified circulatory disorders as the commonest cause of death 43/1000 (95%CI: 30 - 59) followed by tuberculosis and trauma.^14^ In our description, ACS and chronic cardiovascular diseases (CVD) have been predominant causes of deaths with hypertension as a significant common co-morbid condition, a figure which conforms well with local^14^ other Asian statistics.^18^,^19^ This finding has clinical significance as it highlights the impact of cardiovascular disease on morbidity and mortality and the need for interventions to target chronic hypertension.^18^

Clinical severity at presentation has high correlation with mortality and patients who were triaged P1 with high acuity and were unresponsive, had adverse outcomes, consistent with data out of other LMIC.^20^ We found that patients with no history of prior care were critically ill and high priority (P1) cases similar to an Ethiopian study that reported 82.5 % deaths in patients, visiting the ED for the first time in 30 days.^17^

In our study a delayed presentation of >24 hours and a mean length of stay before death in ED of approximately three hours was noted. Crucial time is lost due to limited resources in terms of essential equipment and physician knowledge at primary and secondary health facilities.^9^ This delayed presentation may be compounded by low health literacy, lack of pre hospital care, belief in traditional healers and quality medical facilities inaccessible to the less privileged.^13^,^21^ Over half the mortalities in low-resource settings may be addressed by improved emergency care systems.^22^ Our study has identified that encouraging timely presentation to healthcare, availability of pre hospital care and swift transfer to tertiary care may mitigate some deaths.^13^,^21^ This would require involvement of all stakeholders and collective change in mindset along with development and implementation of legislature.

TIH, ED is post graduate training institute that provides acute care to over 400 patients / day. The 22-bed department has an average of five doctors working in each shift, running two 12-hour shifts. Each shift is supervised by a team lead and day shifts are supervised by consultants. In our study, there was consistency in mortality over 24 hours period in patients of P1 and P2 acuity. Lower acuity patients had a statistically insignificant, high mortality rate in evening and night, probably attributed to limited physical presence of consultants in the evening and night shifts. Similar results have been documented by LMIC and high income countries.^23^,^24^ This warrants a redistribution of hospital resources across all time periods of the day.

### Limitations of the study:

The large sample size in our study has the potential to assess the true mortality at TIH. However, 16.9% of patients either LAMA/DOR or were referred out to other facilities. This along with unknown disposition and incomplete documentation, has limited our analysis and diluted the denominator.

### Recommendations:

Understanding the demographics of mortality through our audit, identifying the gaps and high lighting health system deficiencies can lead to better patient outcome in terms of morbidity and mortality. Public health interventions to improve primary and secondary healthcare and decrease the burden of chronic medical conditions, may ameliorate some of these deaths. The authors hope that this study will serve as a guide to improve the emergency care systems across Pakistan.

## CONCLUSION

The mortalities at TIH ED were on average older, more likely to be male, with delayed presentation, lack of prior medical care and high triage acuity scores. The need to incorporate certain surgical/medical specialties and subsequent reduction of post resuscitation referrals is imperative. This highlights the need to expand our service line and our infrastructure to meet the demands of patient care, hopefully leading to better outcomes. Our study confirms experiences in other LMICs regarding demographics of mortality and has identified the need to develop further audit protocols to elucidate the mortality of P3 acuity patients.

### Author’s contribution:

**SGS and SA:** Conceptualization of project and research guidance.

**SM and AY:** Manuscript writing and literature search, takes responsibility for accuracy and integrity of research.

**SAK and SM:** Data analysis and results write up.

**SM, SAK, SA and AY:** Data analysis, proof reading and editing.
